# Which immunotherapy product is better for patients allergic to Polistes venom? A laboratory and clinical study

**DOI:** 10.1371/journal.pone.0180270

**Published:** 2017-07-07

**Authors:** Eleonora Savi, Cristoforo Incorvaia, Elisa Boni, Marina Mauro, Silvia Peveri, Valerio Pravettoni, Oliviero Quercia, Federico Reccardini, Marcello Montagni, Laura Pessina, Erminia Ridolo

**Affiliations:** 1 Allergy Dept. Unit, G. Da Saliceto Hospital, AUSL, Piacenza, Italy; 2 Cardiac/Pulmonary Rehabilitation, ASST Gaetano Pini/CTO, Milan, Italy; 3 Allergy Unit, Sant’Anna Hospital, ASST Lariana, Como, Italy; 4 Clinical Allergy and Immunology Unit, Foundation IRCCS Ca' Granda Ospedale Maggiore Policlinico, Milan, Italy; 5 Unità ad Alta Specializzazione di Allergologia, Ospedale di Faenza (RA), Faenza, Italy; 6 Azienda Sanitaria Universitaria Integrata Udine, SOC Pneumologia Fisiopatologia Respiratoria, Udine, Italy; 7 Department of Medicine and Surgery, University of Parma, Parma, Italy; Monash University, AUSTRALIA

## Abstract

**Background:**

Venom immunotherapy (VIT) is highly effective in preventing allergic reactions to insect stings, but the appropriate venom must be used to achieve clinical protection. In patients with multiple positive results to venoms, molecular allergy diagnostics or CAP-inhibition may identify the causative venom. Concerning allergy to venom from *Polistes spp*. it has been proposed that only the European species *P*. *dominulus* should be used for VIT. However, this recommendation is not present in any international guideline. Using both laboratory and clinical data, we aimed to evaluate the reliability of this proposal.

**Methods:**

We performed an *in vitro* study using CAP-inhibition to determine sensitization of 19 patients allergic to *Polistes* venom. The clinical study included 191 patients with positive tests to *Polistes* treated with VIT, 102 were treated with *P*. *dominulus* and 89 were treated with a mix of American *Polistes* (mAP).

**Results:**

The difference in % of inhibition was significant concerning inhibition of *P*. *dominulus* sIgE by *P*. *dominulus* venom (79.8%) compared with inhibition by mAP venom (64.2%) and not significant concerning the inhibition of mAP sIgE by *P*. *dominulus* venom (80.1%) and by mAP venom (73.6%). Instead, the clinical protection from stings was not statistically different between the two kinds of venom.

**Conclusion:**

The data from CAP inhibition would suggest that the choice of either *P*. *dominulus* venom or mAP venom for VIT is appropriate in patients with CAP inhibition higher than 70%, but the clinical data show the same odds of protection from stings using for VIT P. dominulus or mAP venom.

## Introduction

Venom immunotherapy (VIT) is an effective treatment for preventing anaphylactic reactions to Hymenoptera stings [1–3]. The choice of venom to be used for VIT is of obvious importance in warranting the clinical protection from the stings of the culprit insect. This is particularly true for patients with multiple positive results to diagnostic tests with venoms and especially for patients allergic to vespids [4]. Because IgE responses to cross-reacting allergens cause positive results to all venoms, comparing the sensitivity of tests to different venoms does not resolve the issue. Previously, it was common to prescribe VIT for all venoms eliciting a positive response, but in recent years, in vitro techniques that can identify the causative venom have been introduced. The first method was RAST-inhibition, by which Hamilton et al. demonstrated that one third of 305 patients with allergic reactions to stings and who tested positive for *Vespul*a- and *Polistes*- IgE in the skin and/or serum were identified as candidates for exclusion of *Polistes* from immunotherapy because their anti-*Polistes* IgE was more than 95% cross-inhibitable with *Vespula* venom [5]. Over the previous decade, molecular allergy techniques have a further advanced, enabling measurement of IgE specific to single venom allergen molecules, thus distinguishing simple cross-reacting components from causative molecules [6]. Three studies showed that by measuring sera from patients with double positivity to *Vespula* and *Polistes* specific IgE to major allergens such as Ves v 1 and Ves v 5 from *Vespula species* and Pol d 1 and Pol d 5 from *Polistes species*, the responsible vespid was identified [7, 8, 9]. In addition, a particular issue was raised in Europe concerning cross-reactivity between the European and the American species. European species, particularly those in Italy, include *P*. *gallicus*, *P*. *dominulus*, *P*. *nymphus*, *P*. *biglumis*, *P*. *associus*, *P*. *sulcifer*, *P*. *semenowi*, and *P*. *atrimandibularis; P*. *gallicus and P*. *dominulus* are the most medically important. American species include *P*. *annularis*, *P*. *fuscatus*, *P*. *metricus*, *P*. *apaches* and *P*. *exclamans*. Cross-reactions among venoms from different *Polistes spp*. are quite extensive [4]. However, in a an evalutation of 21 Italian patients with reactions to *Polistes* stings as determined using skin tests, RAST and RAST inhibition (on only 10 patients), Severino et al. reported that RAST inhibition demonstrated only partial cross-reactivity between American and European species and that *P*. *dominulus*, and *P*. *gallicus*, have exclusive allergens, thus suggesting that such venoms are more suitable than the American mix in Italian patients [10]. However, no other investigations have since been reported. Therefore, we compared venom from *P*. *dominulus* and from the mix of American Polistes (mAP) using *in vitro* CAP-inhibition for diagnosis and retrospectively evaluated the rate of clinical protection from subsequent stings in a group of Italian patients with allergic reactions to *Polistes* venom previously treated with *P*. *dominulus* or mAP, respectively.

## Methods

### Patients

Nineteen patients (15 males, 4 females, age range 16–75 years, mean age 45.9 years) with systemic reactions to Hymenoptera stings of at least Mueller grade II [3], positive skin tests results to *Polistes* venom, and who had not previously been treated by VIT (to avoid treatment-induced changes in specific IgE), were included in the *in vitro* study, which aimed to assess the level of cross-reactivity to *P*. *dominulus* and mAP. A result from skin tests or CAP system showing monosensitization to *P*. *dominulus* was an exclusion criterion.

### In vivo tests

Skin tests were performed by *P*. *dominulus* venom from Anallergo (San Piero a Sieve, Florence, Italy) and mAP venom from Stallergenes (Antony, France), by an initial prick test at 100 mcg/ml, followed, if negative, by intradermal testing at 0.1 mcg/ml and 1 mcg/ml.

### In vitro tests

Sera from all patients were analyzed using CAP inhibition to determine sensitization, following the method previously described by Caruso et al. [7], Savi et al [9)] and Straumann et al [11]. Briefly, two 100 μL aliquots of serum were incubated separately for 12 h at 4°C with 200 mL of *P*. *dominulus* or mAP venom at increasing dilutions (0 μg/mL; 25 μg/mL; 50 μg/mL; 100 μg/mL; 200 μg/mL). Subsequently, specific IgE values (sIgE) against each of the venoms were determined in the prepared samples [9]. The extent of homologous (blockage of venom-specific IgE by the same venom) and heterologous (blockage of the venom-specific IgE by the other venom) inhibition was computed using the following formula: % inhibition = 100 - [IgE inhibited sample (kU/L) x 100/IgE anti-venom (kU/L) at zero concentration of venom]. Serum samples that produced an homologous inhibition of less than 70% were excluded from evaluation (11). Heterologous inhibition >70–75% was considered to be strongly suggestive of cross-reactivity among *P*. *dominulus* and mAP venoms.(9) The protocol used for CAP inhibition was deposited in protocols.io with the identifier dx.doi-org/10.17504/protocols.io.ht7b6rn.

The venom preparations tested were the same as those used for the skin tests. Owing to the limited number of patients, the level of the inhibition obtained with *P*. *dominulus* and mAP were statistically compared using the non-parametric Wilcoxon test.

### Venom immunotherapy

For the clinical part of the study, 191 patients (138 males, 53 females, age range 10–82 years, mean age 48.5 years) with positive tests results to *Polistes* venom prior to VIT and subsequently restung by an identified insect were retrospectively analyzed to determine the rate of protection from *Polistes* venom. Monosensitization to P. dominulus was an the exclusion. Of the 191 patients, 89 were treated with mAP and 102 were treated with *P*. *dominulus* venom. All patients were instructed to recognize *Polistes spp*, and distinguish it from *Vespula spp*., based on anatomical characteristics. In particular, the wasp waist, e.g. the spindle-shaped morphology of the thorax-abdomen joint, is typical of *Polistes spp*, while in *Vespula* and *Vespa spp* this joint is truncated. Only stings from *Polistes spp*. were considered in the analysis. The difference in the rate of protection from stings were analyzed using the chi square test.

All participants or parents/tutors for patients of pediatric age gave their written informed consent.

## Results

The rationale of the *in vitro* study was to assess the relative importance of allergens from *P*. *dominulus* and mAP by measuring their cross-inhibition. Table 1 reports the results of CAP inhibition in individual patients. The mean value of sIgE to *P*. *dominulus* before inhibition was 9.85 ± 8.76 kU/L. After inhibition with *P*. *dominulus* and mAP the values decreased to 1.86 ± 2.08 and 2.80 ± 2.72 kU/L, respectively. The mean value of sIgE to mAP before inhibition was 6.90 ± 7.71 kU/L. After inhibition with mAP and *P*. *dominulus* the values decreased to 1.45 ± 1.60 and 1.35 ± 1.76, respectively. The difference in % of inhibition was significant (p = 0.003) concerning inhibition of *P*. *dominulus* sIgE by *P*. *dominulus* venom (mean% of inhibiton: 79.8%) compared with inhibition by mAP venom (mean % of inhibiton: 64.2%) and not significant concerning the inhibition of mAP sIgE by *P*.*dominulus* venom (mean % of inhibiton: 80.1%) and by mAP venom (mean % of inhibiton: 73.6%). (S1 Fig).

**Table 1:** Results of CAP-inhibition by *Polistes dominulus* and mix of American *Polistes* (mAP) in sera from single patients.

AGE/SEX	IgE Polistes Dominulus (kU/l)	IgE mAP (kU/l)	sIgE to Polistes dominulus after inhibition with Polistes dominulus venom	sIgE to mAP after inhibition with Polistes dominulus venom	sIgE to Polistes dominulus after inhibition with mAP venom	sIgE to mAP after inhibition with mAP venom	
55/F	26,40	25,90	6,9 (74%)	6,14 (77%)	4,53 (83%)	3,5 (86%)	
67/M	0,78	0,46	0,23 (71%)	0,13 (71%)	0,47 (38.8%)	0,11 (75%)	
18/F	4,20	3,70	0,63 (85%)	0,55 (85%)	0,84 (80%)	0,74 (80%)	
42/M	19,04	2,80	2,71 (86.1%)	0,53 (81%)	9,5 (50.7%)	0,78 (72%)	
68/F	16,60	3,80	0,99 (94%)	0,3 (92%)	6,30 (62%)	0,53 (86%)	
41/M	9,80	9,70	1,17 (88%)	1,16 (88%)	0,58 (94%)	0,58 (94%)	
32/M	0,75	0,70	0,16 (78%)	0,06 (91.6%)	0,37 (50%)	0,11 (84%)	
64/M	8,90	6,15	1,62 (82%)	0,92 (85%)	2,88 (68%)	1,85 (70%)	
16/M	12,80	8,53	3,20 (75%)	2,38 (72%)	3,84 (70%)	2,13 (75%)	
75/M	17,50	19,20	2,72 (84%)	2,66 (86%)	4,42 (74%)	4,75 (75%)	
45/F	1,87	1,77	0,28 (85%)	0,14 (92%)	0,94 (49%)	0,69 (54%)	
56/M	0,41	0,33	0,09 (78%)	0,08 (75%)	0,18 (55%)	0,13 (60%)	
26M	1,37	0,97	0,68 (50%)	0,43 (55%)	0,79 (42%)	0,53 (45%)	
68 M	3,38	4,15	0,7 (79%)	0,83 (80%)	1,52 (56%)	1,61 (61%)	
32 M	5,55	5,70	0,44 (92%)	0,45 (92%)	0,44 (91%)	0,45 (91%)	
45 M	25,00	23,00	4,74 (81%)	5,52 (76%)	6,25 (75%)	5,75 (75%)	
42 M	9,80	9,90	1,56 (84%)	1,98 (80%)	1,96 (80%)	1,95 (81%)	
49 M	1,60	1,20	0,30 (81%)	0,26 (78%)	1,12 (30%)	0,42 (65%)	
31 M	21,00	3,10	6,3 (70%)	1,05 (65%)	6,3 (70%)	0,93 (70%)	
	9,83	6,90	1,86	1,35	2,8	1,45	means (kU/l)
	8,74	7,71	2,08	1,75	2,72	1,60	standard deviation
			79,80%	80,10%	64,20%	73,60%	means % inhibition

The rationale of the clinical study was to compare the rate of protection of VIT with *P*. *dominulus* with that of mAP. Incomplete protection from stings, with development of mild to moderate systemic reactions (no life-threatening reaction occurred) was found in 4 of 89 patients treated with mAP (4.5%) and 4 of 102 patients treated with *P*. *dominulus* (3.9%), this difference was not significant. All patients incompletely protected received a doubled maintenance dose (200 μg) and had no reaction when restung. Tables 2 and 3 report the years from starting VIT and the cumulative dose received at the time of the sting. Accordingly, the higher *in vitro* inhibition activity of *P*. *dominulus* compared to that of mAP did not manifest as clinical protection from stings; rather, the clinical protection between the two was comparable.

**Table 2:** Patients treated with *Polistes*. *dominulus* venom.

	Years after VIT initiation and mean cumulative VIT dosage at the time of the sting				
Years from initiation	1 yr (1324 μg)	2 yrs (2272 μg)	3 yrs (3197 μg)	4 yrs (4413 μg)	5 yrs or more (5386 μg)
Pts stung Protected Incompletely protected	21 20 1	37 37 0	25 23 2	10 10 0	9 8 1

**Table 3:** Patients treated with mAP venom.

	Years after VIT initiation and mean cumulative VIT dosage at the time of the sting				
Years from initiation	1 yr (1260 μg)	2 yrs (2276 μg)	3 yrs (3096 μg)	4 yrs (4397 μg)	5 yrs or more (5013 μg)
Pts stung Protected Incompletely protected	29 28 1	35 34 1	12 11 1	5 0	8 1

The clinical study was registered with the Eudract number 2015-003081-92.

## Discussion

Hymenoptera belonging to *Polistes spp* have a worldwide distribution, with variable presence in different areas. In America the most common species are *P*. *annularis*, *P*. *apachus*, *P*. *exclamans*, *P*. *fuscatus*, and *P*. *metricus*, while in Europe *P*. *gallicus* and *P*. *dominulus* prevail. In particular, *P*. *dominulus* is the dominant species in Southern Europe [12]. However, the entomological importance of *Polistes spp*. must be distinguished from the allergological importance. Extensive cross-reactivity is known to occur for *Polistes* allergens, including the species-specific antigen 5, wish shows an 85% identity the European species *P*. *gallicus* and the American species *P*. *annularis* [13]. Furthermore, a consensus documents lack a recommendation to use exclusively *P*. *dominulus* venom for VIT in *Polistes* allergic patients in Europe.

### Comparing the observations from studies on P. dominulus in Italy

In this study we found that in sera from patients with allergic reactions to *Polistes* stings the CAP-inhibition obtained with *P*. *dominulus* was higher than that obtained with mAP. Namely, it was 79.8% for sIgE *P*. *dominulus* with the homologous venom vs. 64.2% with the mAP (p <0.01), and 73.6% for the sIgE mAP with the homologous venom vs 80.1% with *P*. *dominulus* (n.s.). These differences are lower than those reported by Severino et al, who found a value using RAST inhibition of 80.9% on average with *P*. *dominulus*, whereas the inhibition by with mAP was 46.1% on average [10]. To estimate the reliability of these findings we must consider two important issues. First, the number of patients was low (19 in our study and 10 in the study by Severino et al), thus, the results could be stochastic. Indeed, it is quite surprising that observations based on only 10 patients have led to the suggestion to use exclusively *P*. *dominulus* for VIT in patients allergic to *Polistes* venom.

### International knowledge on Polistes venom.

The consensus document from the European Academy of Allergy and Clinical Immunology (EAACI) on Hymenoptera venom allergy stated that the cross-reactivity among European species of *Polistes* (*P*. *dominulus*, *P*. *gallicus*) was stronger than that between European and American species, but there was no recommendation to use only *P*. *dominulus* venom [4]. Second, the importance of the data from RAST- or CAP-inhibition has to must be measured against clinical data. Before *P*. *dominulus* venom became available for VIT in Europe, all patients were treated using mAP with no report of complete treatment failure, i.e. fatal reactions to stings; therefore, the suggestion to use only *P*. *dominulus* venom is flawed. In addition, *P*. *dominulus* is present in the United States [14] and also in this country no fatal reaction has been reported in patients treated with VIT by mAP venom, which is the only *Polistes* venom available. The rate of incomplete protection, i.e. to have a systemic reaction to a sting following VIT, depends upon the kind of venom. Muller et al reported that honeybee VIT provided complete protection in 77% of patients compared with 91% in patients treated with *Vespula spp*. VIT [15]. More recently, better results were observed in a multicenter survey in Europe, with a rate of complete protection of 89% with honeybee VIT and 96% with vespid VIT; however, for vespids, there was no distinction between *Vespula spp*. and *Polistes spp*. [16]. In the present study, we evaluated the rate of complete protection from stings in patients allergic to *Polistes* venom, distinguishing *P*. *dominulus* VIT from mAP VIT. The results showed no statistically significant difference, based on a rate of incomplete protection of 3.9% and 4.5%, respectively, with no life-threatening reaction. This is comparable to the 4% rate of incomplete protection for vespids reported by Rueff et al, though they did not separately analyze *Vespula* and *Polistes* stings [16]. Again surprisingly, in the study by Severino et al. on 104 patients monosensitized to *P*. *dominulus* and treated by VIT when Polistes was recognized as the stinging insect, 11 (10.6%) had systemic reactions, two of which were severe (one grade III and one grade IV) [17].

These contrasting data should stimulate discussion regarding the allergens from *Polistes* venom. The first study dates back to 1982,; when studying sera from 62 American patients allergic to *Polistes* venom, four species' venoms were more closely related to each other than various *Vespula* spp were related to each other. The authors concluded that the commercial *Polistes* venom mix appeared to contain all of the relevant allergens [18]. In the following years the major allergens were identified as hyaluronidase, phospholipase A and B and antigen 5, all showing variable levels of cross-reactivity [19–20]. Concerning *Polistes spp*, these allergens are classified as follows: phospholipase A1 is Pol a 1 (from *P*. *annularis*) and Pol d 1 (from *P*. *dominulus*), hyaluronidase is Pol a 2, and antigen 5 is Pol a 5 and Pol d 5. Hyaluronidase is the main responsible of the cross-reactivity among venom from different species, including not only other vespids but also *Apis mellifera*, while antigen 5 is considered to be species-specific [21]. However, in a study that determined the complete amino acid sequence of antigen 5, the alignment of antigen 5 from the European species *P*. *gallicus* and the American species *P*. *annularis* showed an 85% identity. The sequence identity increased to 98% within the same subgenus, suggesting that the presence of specific epitopes on antigen 5 should be related to the variations on the superficial loops [13]. Remarkably, although antigen 5 was identified more than 30 years ago and investigated in a number of studies, its protein nature and biological role had not yet been ascertained. Of relevant interest, in the venom from the wasp *Polybia scutellaris*, which belongs to the *Polistinae* subfamily, but does not cause allergic reactions, the amino acid analysis of antigen 5 showed a close similarity with antigen 5 of vespids from different species. However, two forms of antigen 5 were isolated, with the amino acids residues at positions 5 and 11 in *P*. *scutellaris* antigen 5 differing from those of the previously known sequences for antigen 5, suggesting that one or the other might be responsible for the lack of allergenicity of the *P*. *scutellaris* venom [22]. Vinzon et al. reappraised the issue, demonstrating that in mice Poly s 5 from *P*. *scutellaris* induced IgG antibodies which cross-reacted with Pol a 5, while Poly s 5 induced only minimal amounts of IgE and was a poor inducer of basophil-mediator release. This finding led the authors to propose Poly s 5 as a candidate for VIT [23], but it should also stimulate investigations on the grade of allergenicity of antigen 5 from more common *Polistinae* including *P*. *dominulus*. Indeed, a different allergenicity of antigen 5 from *Polistes spp*. could be a possible factor explaining the different clinical outcome observed with *Polistes* VIT. This is similar to what is known for food allergy; for example, a divergent allergenicity of apple strains mainly depended on different expression levels of the major allergen Mal d 1, with a proline residue in position 111 causing a drastic reduction of allergenicity [24].

### Conclusions

Currently, there is no scientific report supporting the use of only *P*. *dominulus* for VIT in patients allergic to *Polistes* venom, unless the patient is monosensitized to the latter. In patients with positive test results to both *P*. *dominulus* and mAP, performing VIT with any of the two venoms has the same odds of clinical success.

## Supporting information

S1 Fig Homologous and cross-CAP inhibition by *Polistes dominulus* and mix of American *Polistes*: means of inhibition values. (DOCX)
