# Supplementary material for: Which immunotherapy product is better for patients allergic to Polistes venom? A laboratory and clinical study
Source: PLoS One. 2017 Jul 7;12(7):e0180270. doi: 10.1371/journal.pone.0180270 (PMC5501507; doi:10.1371/journal.pone.0180270)

Figure 1: Homologous and cross-CAP inhibition by *Polistes dominulus* and mix of American *Polistes*: means of inhibition values.


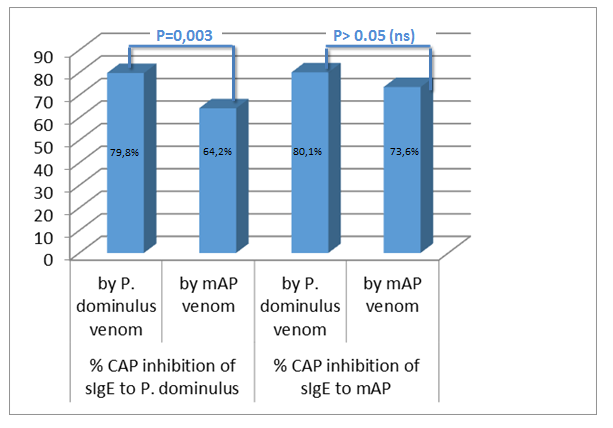

Supplement: S1 Fig — (DOCX) [file pone.0180270.s001.docx]
